# Feasibility of safe outpatient treatment in pediatric patients following intraventricular radioimmunotherapy with 131I-omburtamab for leptomeningeal disease

**DOI:** 10.21203/rs.3.rs-3969388/v1

**Published:** 2024-02-28

**Authors:** Kavya Prasad, Brian E. Serencsits, Bae P. Chu, Lawrence T. Dauer, Maria Donzelli, Ellen Basu, Kim Kramer, Neeta Pandit-Taskar

**Affiliations:** Memorial Sloan Kettering Cancer Center; Memorial Sloan Kettering Cancer Center; Memorial Sloan Kettering Cancer Center; Memorial Sloan Kettering Cancer Center; Memorial Sloan Kettering Cancer Center; Memorial Sloan Kettering Cancer Center; Memorial Sloan Kettering Cancer Center; Memorial Sloan Kettering Cancer Center

**Keywords:** Brain metastases, radiopharmaceutical, radiation safety

## Abstract

**Background:**

Radiolabeled antibody ^131^I-omburtamab was administered intraventricularly in patients with leptomeningeal disease under an institutionally approved study (#NCT03275402). Radiation safety precautions were tailored for individual patients, enabling outpatient treatment based on in-depth, evidence-based recommendations for such precautions. The imperative advancement of streamlined therapeutic administration procedures, eliminating the necessity for inpatient isolation and resource-intensive measures, holds pivotal significance. This development bears broader implications for analogous therapies within the pediatric patient demographic.

**Methods:**

Intraventricular radioimmunotherapy (RIT) with 925–1850 MBq (25–50 mCi) of ^131^I-omburtamab was administered via the Ommaya reservoir, in designated rooms within the pediatric ambulatory care center. Dosimeters were provided to staff involved in patient care to evaluate exposure during injection and post-administration. Post-administration exposure rate readings from the patient on contact, at 0.3 m, and at 1 m were taken within the first 30 minutes, and the room was surveyed after patient discharge. Duration of radiation exposure was calculated using standard U.S. Nuclear Regulatory Commission (US NRC) regulatory guidance recommendations combined with mean exposure rates and whole-body clearance estimates. Exposure rate measurements and clearance data provided patient-specific precautions for four cohorts by age: < 3 y/o, 3–10 y/o, 10–18 y/o, and 18+.

**Results:**

Post-administration exposure rates for patients ranged from 0.16–0.46 μSv/hr/MBq at 1 ft and 0.03–0.08 μSv/hr/MBq at 1 m. Radiation exposure duration ranged from 1–10 days after release for the four evaluated cohorts. Based on the highest measured exposure rates and slowest whole-body clearance, the longest precautions were approximately 78% lower than the regulatory guidance recommendations. Radiation exposure to staff associated with ^131^I-omburtamab per administration was substantially below the annual regulatory threshold for individual exposure monitoring.

**Conclusion:**

^131^I-omburtamab can be administered on an outpatient basis, using appropriate patient-based radiation safety precautions that employ patient-specific exposure rate and biological clearance parameters. This trial is registered with the National Library of Medicine’s ClinicalTrials.gov. The registration number is NCT03275402, and it was registered on 7 September 2017. The web link is included here. https://clinicaltrials.gov/study/NCT03275402.

## BACKGROUND

Neuroblastoma is a rare form of pediatric cancer of neural crest origin, often metastatic to the central nervous system or the leptomeninges [1–3]. Intracavitary treatments have been successfully employed, and administration via the Ommaya reservoir treats the central nervous system/leptomeningeal disease [4, 5].

^131^I-omburtamab is a radiolabeled monoclonal antibody (mAbs) that binds to the B7H3 antigen expressed on the tumor cell membrane. Beta emissions of ^131^I, with a maximum energy of 606 keV (abundance of 89%), deliver radiation to the tumor cells, causing DNA damage and cell death [6–9]. While the high-energy, low-range beta particles are the primary source of treatment, the 364 keV gamma ray emitted by ^131^I (81%) is the primary source of radiation exposure to other individuals, including staff, family members, and the general public [8]. The safety profile was determined in a phase 1/2 study of ^131^I-omburtamab (previously 8H9) [1, 10], with doses of 74 MBq (2 mCi) of ^124^I or ^131^I-omburtamab for dosimetry and 370–2960 MBq (10–80 mCi) of ^131^I-omburtamab for therapy, with a recommended phase 2 therapy dose of 1850 MBq (50 mCi) [8, 10–13]. A follow-up multicenter study was conducted with ^131^I-omburtamab in patients with leptomeningeal disease (NCT03275402).

We established and implemented specific radiation safety procedures to enable the administration of intra-Ommaya radioimmunotherapy treatment on an outpatient basis in non-leaded outpatient rooms while maintaining compliance with federal, state, and local regulations. We compared individualized radiation safety precautions, instructions, and parameters based on the recommendations of the U.S. Nuclear Regulatory Commission (US NRC) Regulatory Guide 8.39 to those generated by incorporating exposure rate and clearance data available from monitoring patients following administration of the treatment doses [14].

Here we present the results of the patient-specific instructions provided to parents, caregivers, and potential visitors (or members of the public) and recommendations for combined use of exposure rates and whole-body clearance [15]. We discuss practical and programmatic components for implementing and evaluating exposure and precautions for patients, healthcare providers, and caregivers.

## METHODS

### Study Design

This manuscript evaluated retrospective data while collecting new information for a prospective study conducted under an approved institutional review board study. The need for informed consent was waived.

### Patients

Exposure rate data for 76 patients was analyzed; of these, 11 patients were treated with an activity of 1221 MBq (33 mCi) and 65 were treated with 1850 MBq (50 mCi). The mean age was 9.8 years (range: 6 months to 18 years).

### Radiopharmaceutical Administration Room Preparation

Patients were treated in MSK’s Pediatric Ambulatory Care Center in preferred rooms in accordance with guidelines for exposure and occupancy. We defined preferred rooms as single-occupancy, corner rooms that shared walls with low-occupancy areas such as stairwells, hallways, and storage rooms, an approach that has been previously described [16]. These rooms were located sufficiently away from public areas such as waiting rooms, play areas, reception desks, and adjacent rooms, with dose rate measurements under 20 μSv in any hour. Before treatment, each room was prepared by radiation safety staff with a waterproof floor covering (polyvinyl chloride PVC), lined trash receptacles, a spill kit, and a radioactive area posting on the door. Following the discharge of the patient, the rooms were surveyed using a Ludlum pancake probe to ensure that contamination levels were below 1000 disintegrations per minute (dpm) over an area of 100 cm^2^, and that all waste was cleared before the room was released back to the unit [16].

### Pre-administration Consultation

Health physicists consulted with each patient’s caregivers before treatment, informing them of post-treatment radiation precautions. Instructions were provided regarding maintaining distance, avoiding close contact, and being conscious of bodily fluids. For the relatively younger patient population (< 3 y/o), additional and more involved care was anticipated, including, but not limited to, feeding, changing, and disposal of contaminated diapers and the presence of younger siblings at home. Training was provided to parents and caregivers to assist their child appropriately, detailing care involved in diaper changes, feeding, bathing, and other day-to-day activities while minimizing time spent very close to them, acknowledging that unexpected needs may arise that require more involved care. Electronic dosimeters (Isotrak) were provided to caregivers for the duration of their stay following treatment to study exposure of an individual sitting in the room.

### Dose Administration

The radioimmunotherapy dose was administered under aseptic conditions accessed by trained physicians or nurse practitioners in coordination with the nuclear medicine physician and authorized user. A health physicist from the radiation safety service was present to supervise radiation safety aspects during the administration.

### Measurement of Radiation Exposure

Measurements from the patient were taken within the first 30 minutes post-injection with a 451B (Fluke Biomedical) ion chamber with the beta window closed for a more accurate energy response to iodine due to the slight over-response for the 364 keV emission with the window open [17]. Measurements were taken on contact with the administration site, at 30 cm, and at 100 cm, in direct line of sight from the injection site or Ommaya reservoir—most often behind the head while the patient was in a supine position, to limit self-shielding and radiation scattering from the patient.

Evaluations for staff and caregiver exposure levels during and post-administration were obtained from electronic dosimeters (DMC 3000 Mirion) worn on the main torso facing the patient to measure deep dose equivalent (DDE) exposure to the whole body. This included the primary registered nurse who cared for the patient for the rest of their stay. Staff involved in administering the drug were also given two extremity monitors (ring dosimeters Saturn TLD ring (Mirion), one on each hand). Whole body data was collected for 16 administrations and computed for the entire population through computation of measured exposure rates per unit activity and mean recorded exposure on the dosimeters, illustrated in the example below, where dosimeter data from Patient A is known and used to evaluate estimated dose to staff from Patient B:

ReadingonelectronicdosimeterforstafffromknownPatient A:50μSv

$$\text{Measured}\;\text{exposure}\;\text{rate}\;\text{per}\;\text{unit}\;\text{activity}\;\text{administered}\;\text{for}\;\text{Patient}\;\;A:100\frac{\mu Sv}{hr}\;\text{per}\;1850\;\text{MBq}=0.054\frac{\mu Sv}{hr-\text{MBq}}$$

$$\text{Measured}\;\text{exposure}\;\text{rate}\;\text{per}\;\text{unit}\;\text{activity}\;\text{from}\;\text{Patient}\;\text{B : }350\frac{\mu Sv}{hr}\;\text{per}\;925\;\text{MBq}=0.38\frac{\mu Sv}{hr-\text{MBq}}$$

$$\text{Estimated}\;\text{dose}\;\text{to}\;\text{staff}\;\text{caring}\;\text{for}\;\text{patient}\;\text{B }=\frac{0.38\times 50}{0.054}=350\mu Sv$$

Exposure data was further analyzed for different activity levels (925 MBq, 1221 MBq, and 1850 MBq) and corresponding patient age (0–1 y/o, 1–3 y/o, 3–10 y/o, and 10–18 y/o) cohorts to estimate expected exposures to staff and caregivers for the wide range of patients that were treated.

### Radiation Exposure Calculations

Family member/visitor exposures were calculated using modeling based on Release Equations 1 (lifetime exposure) described by the US NRC and a resulting modified release Eq. 2 (exposure as a function of time) to use real-time, patient-specific exposure rates and occupancy factors specific to varying day-to-day situations, while keeping annual limit of exposures for caregivers (5 mSv) and members of the public (1 mSv ) in mind [18]. Modified precaution times were modeled on the calculations below where the following situations were evaluated: sleeping apart from vulnerable populations including children and pregnant adults, holding a child in one’s lap, sleeping apart from adults, and maintaining distance from vulnerable populations and members of the public, with an assumed occupancy of 33% for separations of 0.3 m for sleeping, holding a child in one’s lap, bathing, feeding, and other close interactions, and 25% for separations of 1 m involving visitation and interactions in the public [18]. Internal recommendations limited the exposure received to a member of the public to half of the annual limit of exposure for the parent/guardian to allow for multiple treatments to occur, as needed. As Low As Reasonably Achievable (ALARA) guidelines can be chosen differently to allow for varying number of treatments per requirements.


$$D(t)=\frac{34.6\times \Gamma \times Q_{O}\times T_{E}\times \left(1-e^{\frac{-0.693t}{T_{p}}}\right)}{r^{2}}$$

$$D(t)=34.6\times T_{E}\times X\times \left(1-e^{\frac{-0.693t}{T_{p}}}\right)$$

Where,

D(t) = Accumulated exposure at time t, in roentgens

34.6 = Conversion factor of 24 hrs/day times the total integration of decay (1.44)

Γ = Specific gamma ray constant for a point source, R/mCi-hr or μSv/hr-MBq at 1 cm [18]

Q_0_ = Initial activity of the point source in millicuries, at the time of the release

T_e_ = Effective half-life in days

T_p_ = Physical half-life in days

r = Distance from the point source to the point of interest in centimeters

t = Exposure time in days

X = measured exposure rate in mR/hr or μSv/hr at distance r

$\left(1-e^{\frac{-0.693t}{T_{p}}}\right)$= modified occupancy factor

The modified precautions calculated using the method described above were presented to the patient and their caregivers pre-administration. Post-administration measurements were collected using a 451 B (Fluke Biomedical) Ion Chamber within the first 30 minutes and applied to calculations.

Parents and caregivers were given precautions based on patient release equations in Regulatory Guide 8.39. In addition, using biological clearance data from 28 patients (total of 43 cases) from prior published work, precaution times were calculated using a combination of normalized exposure rate measurements in μSv/hr/MBq and biological clearance parameters in hours, herein described as modified precautions (*13–14*). These were then compared to precautions generated using default regulatory guidance, herein described as default precautions [19].

The default precautions are represented as Group 1. Three other scenarios for patient release precautions (i.e., longest, median, and shortest) were generated using the normalized exposure rates and biological clearance data. Group 2 comprised the highest exposure scenario, 95th percentile of clearance (highest retention) and 95th percentile of normalized exposure rate. Group 3 was the median exposure scenario—50th percentile of clearance (median retention) and 50th percentile of normalized exposure rates. Group 4 was the lowest exposure scenario: 5th percentile of clearance (lowest retention) and 5% of normalized exposure rates.

## RESULTS

### Radiation Exposure and Clearance Rates

**Figure 1:**
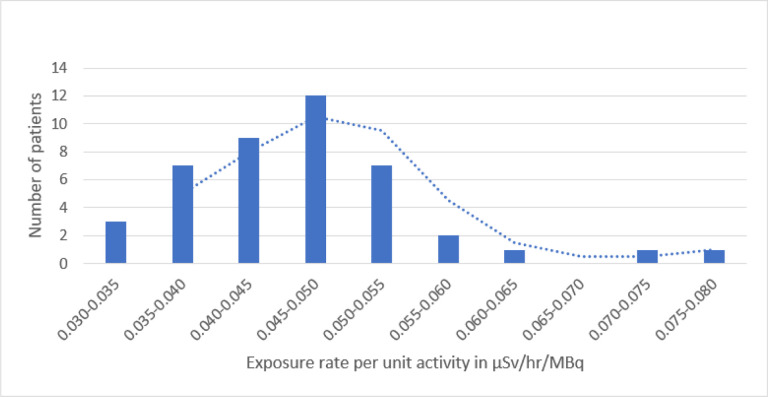
Distribution of normalized exposure rates in μSv/hr/MBq

Radiation exposure rate measurements and biological clearance data from 28 patients—with a total of 43 treatments—were evaluated, representative of our study population. Median exposure rate measurements were 2100 μSv/hr (1060–7200) on contact, 480 μSv/hr (320–880) at 0.3 m, and 87 μSv/hr (58–148) at 1 m. These measurements were further broken down into four cohorts (Groups A, B, C, and D) and evaluated based on prescribed activity (925, 1221, 1850, and 1850 MBq) and patient age (< 1 y/o, 1–3 y/o, 3–10 y/o, and 10–18 y/o), as described in Table 1. Using the normalized dose measurements, whole-body effective clearance values were calculated to be between 35.9–44.2 hours, with a range of 23.5 to 69.5 hours. The distribution of normalized exposure rates has also been illustrated in Fig. 1 in uSv/hr/MBq.

### Exposure to Caregivers

The median measured exposure to a caregiver obtained over a four-hour post-administration period was 85 μSv, with a range of 50 to 140 μSv, collected for 10 cases.

### Exposure Data in Staff

The median measured whole-body exposure for the nurse practitioner was 35 μSv, physician was 27 μSv, authorized user was 5 μSv, health physicist was 12 μSv, and registered nurse was 54 μSv. Mean exposure to the staff was evaluated per cohort and recorded as described in Table 2. Most of the exposures on the ring dosimeters resulted in an “M” or minimal (< 100 μSv) value per administration on the report provided by Mirion. Maximum measured extremity exposure was 760 μSv over a 4.5-minute injection time.

### Algorithm-generated Precaution Times

The algorithm-generated precaution times are based on the radiation exposure calculations in the methods section using patient-specific exposure rates, occupancy factors, and clearance data. We evaluated precaution scenarios based on the regulatory guidance 8.39 represented in Group 1, precautions for the highest exposure and highest retention at the 95th percentile in Group 2, median exposure, and median retention at the 50th percentile in Group 3, and the lowest exposure and lowest retention at the 5th percentile in Group 4. The duration of precaution times based on exposure scenario are evaluated and summarized in Table 3.

## DISCUSSION

The designated treatment rooms are large enough to accommodate the patient, medical equipment, staff, and caregivers, while ensuring that exposure measured outside the room is minimal without the use of lead shielding, and followed regulations of maintaining dose rates in public areas under 20 μSv/hr, achieved by strategically using rooms further away from public or frequently occupied areas. Use of waterproof floor coverings and lined trash bags allowed for all contamination to be properly discarded from the room. Radiation safety postings on the door informed staff about precautions, personal protective equipment requirements, and spill response procedures.

Despite the lack of shielding, the results provided by the electronic whole-body and extremity ring dosimeters demonstrated that staff exposure was minimal, as the duration of close contact was often only a few minutes. Exposure readings of greater than 100 μSv were attributed to unexpected adverse situations such as vomiting, extensive patient care by the nurse, or spill response. Staff responding to adverse situations donned and doffed personal protective equipment and were surveyed by the health physicist for contamination before exiting the room. The electronic whole-body dosimeters also demonstrated that exposure to parents or caregivers during and post-administration of the drug was too low to factor into developing the modified precautions as they did not substantially affect the annual limit of exposure.

All patients were monitored on site for side effects, anaphylaxis, and/or adverse events for four hours post-injection, and then for three days via follow-up appointments with the patient staying nearby.

Use of biological clearance information introduced an additional factor into exposure calculations that had not been previously employed for assessing exposure and release parameters for pediatric patients receiving radioiodine immunotherapies. Guidance provided in Regulatory Guide 8.39 Revision 1 provides a baseline suggestion for precautions to use physical half-life and no patient self-shielding for patient release purposes. If a licensee considers biological clearance or self-shielding for patient release, these factors must be documented and kept with patient release records. Practically, a patient is a very diffused source of radiation with a large percentage excreted by the body through urine, with trace amounts in sweat and saliva. Effective clearance time was thus much shorter than the physical half-life of the material, resulting in shortened precaution times. Even in the highest exposure scenario (highest exposure rate measurements and retention), the resulting modified precaution times were approximately 78% lower than the default precautions recommended.

Patients and their families were able to go home after a few hours in the hospital, which allowed them to resume their lives and be secure knowing that they were just a few minutes away if they needed medical assistance. To be able to receive the treatment and care they needed, and not spend additional time in a hospital, was paramount to their recovery and response to treatment, as evidenced by improved overall survival through several phases of the clinical trial at MSK [18]. The benefits of outpatient versus inpatient have been studied extensively, and the advantages of “cancer outside the hospital walls” can be translated to radioimmunotherapy, and far outweigh the risks [18]. Extensive education, training, and support must be established with staff prior to implementing such a program in its full scale.

## CONCLUSION

Our calculations evaluate exposure to caregivers and staff using patient exposure rate measurements and biological clearance data. These calculations allow for continued treatment with ^131^I-omburtamab on an outpatient basis while meeting all regulatory requirements for patient release. Caregivers can stay with their child during and after treatment, with only modest radiation precautions once released from the hospital. Radiation exposure to staff was found to be minimal and was not a limiting factor in outpatient treatment with ^131^I-omburtamab. With proper staff training on basic radiation safety, such as minimizing time spent in very close proximity to the patient, these treatments can be completed in standard treatment rooms without the need for specialized lead-lined rooms, reducing financial burdens and emotional stressors for patients’ families and institutions alike.

## Figures and Tables

**Table 1:** Distribution of normalized exposure rates in μSv/hr/MBq across the four treatment groups

Group	Prescribed activity	Age	Normalized exposure rate at 1 ft (μSv/hr/MBq) [median (range)]	Normalized exposure rate at 1m (μSv/hr/MBq) [median (range)]	Whole-body effective clearance (hrs.) [median (range)]
A	925 MBq	< 1y	0.28 (0.27–0.32)	0.059 (0.054–0.062)	N/A*
B	1221 MBq	1–3 y	0.38 (0.30–0.46)	0.062 (0.051–0.078)	35.9 (33.9–39.8)
C	1850 MBq	3–10 y	0.27 (0.16–0.46)	0.046 (0.032–0.065)	39.7 (25.8–61.0)
D	1850 MBq	10–18 y	0.27 (0.16–0.43)	0.046 (0.030–0.076)	44.2 (23.5–69.5)

* Whole-body effective clearance was not analyzed for this group

**Table 2:** Measured whole-body exposure data from injecting medical doctor (MD)/nurse practitioner (NP), authorized user (AU), registered nurse (RN), and health physicist (HP)

Group*	Activity	Age	Injecting MD/NP (μSv) [median (range)]	Nuclear Medicine AU (μSv) [median (range)]	Primaiy RN (μSv) [median (range)]	HP (μSv) [median (range)]
B	1221 MBq	1–3 y	30 (26–36)	5 (4–6)	49 (39–59)	0.015 (0.012–0.018)
C	1850 MBq	3–10 y	42 (23–61)	6 (4–9)	69 (39–100)	0.021 (0.012–0.031)
D	1850 MBq	10–18 y	44 (25–67)	7 (4–10)	77 (44–97)	0.024 (0.014–0.030)

* Group A was not evaluated at the time this data was collected.

**Table 3:** Duration of precautions in days for various scenarios based on exposure and biological retention.

Scenario	Group 1	Group 2	Group 3	Group 4
Sleep apart from children and pregnant adults	64	16	7	4
Holding children in lap	59	14	6	3
Sleeping apart from other adults	46	10	4	1
Distance from children, pregnant adults, and members of public	36	8	3	1
Distance from non-pregnant adults	32	7	3	1
Maintaining proper hygiene	46	10	4	1

## Data Availability

Information regarding this study can be found in the US National Library of Medicine’s Clinical Trial Registry. Web link: https://classic.clinicaltrials.gov/ct2/show/NCT03275402. Additional information is available in a prior published study. Web link: https://jnm.snmjournals.org/content/64/6/946.long. The datasets generated during and/or analyzed during the current study are available from the corresponding author on reasonable request.

## Abbreviations

RIT: radioimmunotherapy
US NRC: United States Nuclear Regulatory Commission
μSv: micro sieverts
MBq: mega becquerel
mAbs: monoclonal antibodies
MSK: Memorial Sloan Kettering Cancer Center
kEv: kilo electron volt
mCi: millicurie
PVC: polyvinyl chloride
dpm: disintegrations per minute
y/o: year(s) old
mSv: milli sieverts
ALARA: as low as reasonably achievable
